# The Biology of *Neisseria* Adhesins

**DOI:** 10.3390/biology2031054

**Published:** 2013-07-29

**Authors:** Miao-Chiu Hung, Myron Christodoulides

**Affiliations:** Neisseria Research, Molecular Microbiology, Clinical and Experimental Sciences, Sir Henry Wellcome Laboratories, Faculty of Medicine, University of Southampton, Southampton General Hospital, Southampton, SO16 6YD, UK; E-Mail: m.hung@soton.ac.uk

**Keywords:** *Neisseria*, meningitis, gonorrhoea, bacterial adhesin, host cell receptor, structure

## Abstract

Members of the genus *Neisseria* include pathogens causing important human diseases such as meningitis, septicaemia, gonorrhoea and pelvic inflammatory disease syndrome. *Neisseriae* are found on the exposed epithelia of the upper respiratory tract and the urogenital tract. Colonisation of these exposed epithelia is dependent on a repertoire of diverse bacterial molecules, extending not only from the surface of the bacteria but also found within the outer membrane. During invasive disease, pathogenic *Neisseriae* also interact with immune effector cells, vascular endothelia and the meninges. *Neisseria* adhesion involves the interplay of these multiple surface factors and in this review we discuss the structure and function of these important molecules and the nature of the host cell receptors and mechanisms involved in their recognition. We also describe the current status for recently identified *Neisseria* adhesins. Understanding the biology of *Neisseria* adhesins has an impact not only on the development of new vaccines but also in revealing fundamental knowledge about human biology.

## 1. Introduction

The Genus *Neisseria* (Kingdom Bacteria, Phylum Proteobacterium, Class β-Proteobacterium, Order *Neisseriales*, Family *Neisseriaceae*) includes at least 25 species (Table 1) based on 16S rRNA gene sequence information. *Neisseria meningitidis* and *Neisseria gonorrhoeae* are obligate human pathogens and the other strains are either commensal organisms in humans and mammalian species and/or have been reported to cause opportunistic human infections. The noted Austrian pathologist and bacteriologist Anton Weichselbaum (1845–1920) first identified a *Diplococcus intracellularis meningitidis* from the cerebrospinal fluid (CSF) of patients with “epidemic cerebrospinal meningitis” in 1887 [1]. This organism was later reclassified as a member of the genus *Neisseria*, after the German physician Albert Neisser (1855–1916) who discovered in 1879 the diplococcus known as *Neisseria gonorrhoeae*. In 1884, the Italian physicians and zoologists Ettore Marchiafava (1847–1935) and Angelo Celli (1857–1914) described the presence of oval micrococci within leucocytes in the CSF of patients dying with meningitis [2], and in 1896, the German physicians and microbiologists Johann Heubner (1843–1926) and F. Kiefer were the first to isolate meningococci from the CSF and throat of living patients [3,4,5].

**Table 1:** The Genus *Neisseria*.

Neisseria species		Colonisation sites	Clinical disease
Obligate pathogen	*N. meningitidis*	Nasopharynx	See text for details
	*N. gonorrhoeae*	Urogenital tract	See text for details
Opportunistic	*N. lactamica*	Nasopharynx	Meningitis, septicaemia
	*N. sicca*	Nasopharynx	Endocarditis [6], bacteremia [7], meningitis
	*N. subflava*, which contains previous spp. *N. flava*, *N. perflava* and *N. subflava*	Nasopharynx Urogenital tract	Bacteremia [7], meningitis
	*N. cinerea*	Nasopharynx	Newborn ocular infection
	*N. elongate*, including 3 subspecies: *N. elongate* subsp. elongate, subsp. glycolytica and subsp. nitroreducens.	Nasopharynx	Septicaemia, endocarditis, brain abscess [8]
	*N. flavescens*	Nasopharynx	Meningitis, septicaemia, endocarditis
	*N. mucosa*	Nasopharynx	Pneumonia
	*N. pharyngis*	Nasopharynx	Septic arthritis
	*N. polysaccharea*	Nasopharynx	Not known
	*N. canis*	Isolated from the throats of cats	Cat-bite wound infection
	*N. weaveri*	Normal oral flora in dogs	Dog-bite wound infection
	*N. iguana*	Zoonotic	Not known
	*N. animalis*	Isolated from the throats of guinea pigs	Not known
	*N. denitrificans*	Isolated from the throats of guinea pigs	Not known
	*N. dentiae*	Zoonotic; isolated from dental plaque of domestic cows	Not known
	*N. macac* *ae*	Isolated from the oropharynges of rhesus monkeys	Not known
	*N. zoodegmatis* & *N. animaloris*	Commensal organisms in the oral cavity of dogs and cats	Systemic infections in humans and animals; dog-bite wound infection [9]
	*N. bacilliformis*	Not known	Wound infection, respiratory tract infection [10], endocarditis [11,12]
	*N. skkuensis*	Not known	Isolated from a foot ulcer in a diabetic patient [13]
	N. wadsworthii & *N.* shayeganii	Not known	Wound infection [14]
	*N. oralis*	Healthy subgingival plaque [15]	Not known
False	*N. caviae*, *N. ovis*, *N. cuniculi* [16]		

### 1.1. Meningococcal Disease

*Neisseria meningitidis* (the meningococcus) causes approximately 500,000 cases of infection per year globally and up to 50,000 deaths [17]. The incidence of meningococcal disease ranges from less than 0.2/100,000 to over 1,000/100,000 population/year and the two peak attack rates occur in children less than one year of age, and in adolescents and young adults [18]. The distinguishing features of meningococcal disease are the fulminant clinical course and the ability to cause large-scale epidemics. The French physician Gaspard Vieusseux (1746–1814) is generally credited with the first detailed description of epidemic meningitis in 1805 in the environs of Geneva, with 33 deaths occurring during a three-month period. His cases show classical clinical signs of meningococcal meningitis in children, with rapid onset and death within 24–48 h [19]. Moreover, children presenting without meningeal irritation showed signs of fulminant disease, including violent abdominal pain, vomiting, diarrhoea and the presence of livid spots on the skin. The clinical manifestations of meningococcal disease can be classified into (1) bacteraemia without sepsis; (2) meningococcaemia without meningitis; (3) meningitis with or without meningococcaemia and (4) meningoencephalitis [20,21]. These clinical presentations are not mutually exclusive and often overlap in individual patients and they are more useful as prognostic predictors [20]. Brandtzaeg *et al.* recently proposed a clinical classification system for research purposes of (1) shock without meningitis; (2) shock and meningitis; (3) meningitis without shock; and (4) meningococcaemia without shock or meningitis. This classification has been used in clinical studies of meningococcal disease occurring amongst 862 subjects and a higher mortality rate was observed in patients with shock [22].

The most common presentation of invasive meningococcal disease is meningitis, while fulminant meningococcal septicaemia has a higher mortality rate [23]. Critical cases may develop disseminated intravascular coagulation (DIC) and acute adrenal haemorrhage. In cases with severe meningococcaemia, intravascular thrombosis and haemorrhagic necrosis can cause dramatic widespread *purpura fulminans* with potential infarction and gangrene of limbs [21,23]. Meningococci can also cause pneumonia, which occurs in 5–15% of patients with invasive meningococcal disease [24]. Other syndromes associated with meningococcal disease include acute respiratory distress syndrome (ARDS), conjunctivitis, otitis media, epiglottitis, urethritis, arthritis, pericarditis, *conus medullaris* syndrome and cranial nerve dysfunction, especially of the 6th, 7th and 8th cranial nerves. Severe pericarditis, which is likely to result from an immunological reaction thought to be endotoxin-related, can complicate massive tamponade [21,24,25].

Early administration of antibiotics is the key factor leading to full recovery. Empirically, a third-generation cephalosporin (e.g., cefotaxime, ceftriaxone) should be given once the diagnosis is suspected. Penicillin G is still the drug of choice if the antibiotic susceptibility of the causative meningococcus is known. Alternatively, chloramphenicol can also be effective [26]. Despite the availability of effective antibiotics, the mortality rate remains at 10–15% of all cases [27]. Without treatment, the mortality rate associated with meningococcal disease can be as high as 70–90%. Up to 25% of survivors of meningococcal disease have lifelong sequelae, such as hearing loss, neurological disability (e.g., mental retardation, seizures and cognitive dysfunction), hydrocephalus, renal failure, skin scarring or loss of a limb [24]. However, survivors who do not display gross neurological damage can often show more subtle neurodevelopmental sequelae, e.g., the impairment of cognitive ability and behavioural limitations are factors that can influence academic performance in mathematics, reading and writing [28,29].

### 1.2. Gonococcal Disease

*Neisseria gonorrhoeae* (the gonococcus) is the causative agent of gonorrhoea, which has affected humans for thousands of years and is still a commonly reported sexually transmitted disease (STD) worldwide. Every year, this exclusively human pathogen afflicts an estimated 62 million people [30]. Gonococcal infection can often be asymptomatic and depending on the anatomical site of exposure, clinical infection can be urogenital, anorectal or pharyngeal gonorrhoea. The major symptoms of urogenital infection in men include urethral discharge and dysuria and the most common localized complication is acute epididymitis: other complications include penile lymphangitis, periurethral abscess, acute prostatitis, and seminal vesiculitis. By contrast, the natural course of gonococcal infection is less well understood in women and there are probably more cases of subclinical infection in women than men. The primary site of female genital infection is the mucosal columnar epithelium in the endocervix, whilst the squamous epithelium of the vaginal mucosa is influenced by oestrogen and not susceptible to gonococcal infection. In women, gonococcal infection can cause cervicitis, endometritis or salpingitis (inflammation in the fallopian tube). Complicated ascending gonococcal infection can lead to pelvic inflammatory disease (PID), ectopic pregnancy and infertility in women [31]. In addition, co-infection with gonococci and *Chlamydia trachomatis* is common [32,33] and moreover, gonococcal infection can facilitate transmission of human immunodeficiency virus (HIV) [34].

Other local manifestations include gonococcal conjunctivitis, gingivitis, intraoral and cutaneous abscess formation. Pharyngeal gonococcal infections are mostly asymptomatic and resolve spontaneously. Infants born to infected mothers are at high risk of acquiring gonococcal conjunctivitis (*ophthalmia neonatorum*), which can cause childhood blindness. Gonococcal dissemination through the blood stream, although an uncommon occurrence, can cause “disseminated gonococcal infection” (DGI) and arthritis-dermatitis syndrome is the most common presentation. This arthritis is often asymmetric and involves a few joints, in contrast to polyarthritis, which is caused by immune complex-mediated disorders. Characteristic dermatitis may present as papules and pustules, often with a haemorrhagic component. Occasionally, endocarditis, meningitis, osteomyelitis, septic shock and ARDS are complications of DGI. Although uncommon, direct extension of *N. gonorrhoeae* or *C. trachomatis* from the fallopian tube to the liver capsule and adjacent peritoneum can cause acute perihepatitis (Fitz-Hugh-Curtis Syndrome).

Effective treatment is essential for disease control. However, the increase in antibiotic-resistant gonococcal strains worldwide is worrying and from 2010, the Centerfor Disease Control (CDC) no longer recommends oral cephalosporins for treating gonococcal infections [35]. A combination therapy consisting of a single intramuscular injection dose of ceftriaxone (250 mg) and oral azithromycin (1 g), is essential to slow down the development of drug resistance [36].

Members of the genus *Neisseria* colonise exposed mucosal epithelial surfaces of mammalian species, but as demonstrated above in the pathology of *Neisseria* infections, the ability to disseminate from sites of colonisation also provides the opportunity for bacterial interactions with a wide variety of host cell types and organ systems. In this review, we examine the process of adhesion of *Neisseria* species to target mammalian host cells and tissues, by focusing on the (i) biology and structure of adhesins; and (ii) the mechanisms involved in their interactions. We examine also *Neisseria* surface structures that are involved in adhesion but not defined strictly as adhesins. 

## 2. *Neisseria* Surface Structures Involved in Adhesion

To colonize host cells successfully, *Neisseria* spp. need to both establish and maintain an association with host cell surfaces. The first contact between the bacterium and host cells involves the process of adhesion, which can depend on the interaction of specific bacterial surface molecules—adhesins—with specific host cell receptors. Colonization (or maintenance of association with host cells) involves adhesion, bacterial aggregation, microcolony and biofilm formation and the avoidance of host immunity. To begin, we focus on the biology and structure of *Neisseria* adhesins. 

### 2.1. Pilus

The Type IV pilus (Tfp) is probably the best-studied adhesin of *Neisseria*. Commonly found in Gram-negative bacteria [37], the Tfp imparts twitching motility by rapid extension and retraction [38] and facilitates uptake of foreign DNA to increase transformation frequency [39]. Meningococci are capable of producing two structurally distinct types of pili—Class I and II—whilst gonococci only produce Class I pili. The discriminating murine monoclonal antibody SM1 [40] binds to both meningococcal and gonococcal Class I pili, but not to meningococcal Class II pili [41,42]. Little is known about the expression of Neisserial pili in commensal strains: some strains of *N. lactamica*, *N. flava*, *N. pharynges* and *N. sicca* were non-reactive with SM1 [43] and a comparative analysis of the pilin gene in pathogenic and non-pathogenic *Neisseria* has demonstrated two distinct structural groups, one consisting of the pilin genes from *N. lactamica*, *N. cinerea* and the Class II pilin-producing subset of *N. meningitidis* isolates, and the other consisting of gonococcal and meningococcal Class I pilin-encoding genes [44]. Moreover, *N. sicca*, *N. subflava*, and *N. elongata* were shown to contain two putative *pilE* genes arranged in tandem, whilst *N. polysaccharea*, *N. mucosa*, and *N. denitrificans* harboured only single genes [45]. Recently, the fimbriae of *N. elongata* were identified as Tfp that are capable of mediating horizontal gene transfer with *N. gonorrhoeae* [46]. 

Antigenic variation of Tfp expression, which results from both inter- and intra-genomically non-reciprocal DNA recombination between *pilS* (silent) genes and *pilE* genes [41,47,48,49,50] is known to contribute to evasion mechanisms employed by pathogenic *Neisseria* [51]. Furthermore, pili from different infection sites of the same patient with meningococcal disease can be antigenically diverse [52]. The frequency of antigenic variation was reported to be 0.13 and 0.03 recombination events per cell with a rate of 4 × 10^−3^ and 1.6 × 10^−3^ events per cell per generation for gonococci [53] and meningococci [54], respectively.

Neisserial pili are hair-like, flexible and helically homopolymeric fibres, 6 nm in diameter and several microns in length. PilE (the product of the *pilE* gene) is the pilin subunit that assembles into the multifunctional pilus adhesin and virulence factor [55]. To our knowledge, the first report of the preparation of three-dimensional needle- and plate-shaped crystals of purified *N. gonorrhoeae* pilin (strain MS11 variant C30) was made as long ago as 1987 by Parge and colleagues [56]. The best crystals were diffracted to 2.4 Ǻ (1 Å = 0.1 nm) resolution using synchrotron radiation [57]. In a follow-up paper, Parge and colleagues derived the structure of the fibre-forming pilin protein at 2.6 Ǻ resolution [58]. The crystallographic structure of *N. gonorrhoeae* pilin revealed an α-β roll fold with a 85 Ǻ α-helical spine and an O-linked disaccharide. Key residues were identified that stabilized interactions that allowed sequence hyper-variability, correlating with antigenic variation, within disulphide region β-strands and connections. The surface shape, hydrophobicity and sequence variation of pilin is believed to constrain pilus assembly to the packing of flat subunit faces against α1 helices. A core of coiled α1 helices is banded by β-sheet to form the assembled helical fibre, with carbohydrate and hyper-variable sequence regions exposed outwards to solvent. Further X-ray crystallographic refinement of gonococcal pilin to 2.6 Ǻ resolution, along with mass spectrometry of peptide fragments, revealed the presence of phosphorylated serine at position 68 [59]. Dephosphorylation altered the morphology of fibres, but did not affect bacterial adhesion, transformation, piliation or twitching motility. Parge *et al.* also obtained reassembled pilus fibers and three-dimensional crystals for pilin protein from three gonococcal strains [56], and using synchrotron X-ray radiation (3 Ǻ resolution) they confirmed the “packing” arrangement of the pilin subunits as observed in pilus fibers using electron microscopy (EM). The proposed model is an anti-parallel 4-α helix for the overall polypeptide fold of a pilin subunit. At the same time, cryo-electron microscopy (cryo-EM) and reconstruction provided a structure for gonococcal Tfp, in which spiralling three-helix bundles form the filament core, anchor the globular heads and provide strength and flexibility. Hyper-variable loops were observed to protrude from a “corrugated” pilus surface, which was created by the shielding of conserved functional residues by post-translational modifications in the globular heads [60].

Associated with pilus are 23 proteins, of which 15 (including PilC1/C2, PilD, PilE, PilF, PilG, PilH, PilI, PilJ, PilK, PilM, PilN, PilO, PilP, PilQ and PilW) are essential for Tfp biogenesis, which involves four steps: (1) assembly; (2) functional maturation; (3) counter-retraction; and (4) emergence onto the cell surface [61]. By contrast, seven other proteins (ComP, PilT, PilT2, PilU, PilV, PilX and PilZ) are not necessary for piliation, but are involved in “fine-tuning” Tfp function [62]. The *pilM*, *pilN*, *pilO*, *pilP* and *pilQ* genes are organised as an operon and the pili proteins PilD, PilF, PilM, PilN, PilO and PilP are important for pilus assembly. Recently, PilM, PilN, PilO and PilP proteins have been shown to form a pilus subcomplex that is involved in pilus assembly [63]. PilC, PilI, PilJ, PilK and PilW are related to functional maturation [61]. Furthermore, PilC has been demonstrated to mediate fibre retraction [64]. The *pilG* gene is highly conserved in pathogenic *Neisseriae* [65] and the PilG protein plays a role in pilus retraction. However, PilG does not appear to be essential for pilus assembly, because apparently normal pili are observed in *pilG* mutant meningococci [61]. The conserved OM-localized PilQ is a secretin that forms a pore through which Tfp emerge on the bacterial surface [66] and PilW is important for the stability and function of the pilus fibre [67]. Though low in abundance, PilX plays a role in mediating bacterial aggregation, which is important for bacterial adhesion [68]. Recently, another minor (low abundance) pilin, ComP, was reported to have a binding affinity to DNA uptake sequence (DUS) and therefore contributes to selective DNA uptake during transformation [69].

The first three-dimensional structure of the secretin PilQ was resolved at 2.5 nm [70], and showed that the complex has a 12-fold rotational symmetry and the dominant feature is a 10 nm deep cavity within the centre of the complex. The quaternary structure of the PilQ secretin from *N. meningitidis* was analysed by transmission electron microscopy (TEM) with a negative stain [71], to a resolution of ~2.6 nm and describes a dodecameric quaternary structure. Oligomeric PilQ adopts a “doughnut-like” structure and initial measurements determined that the external ring was 16.5 nm in diameter, surrounding a central cavity that was 6.5 nm in diameter. PilQ is organized as a ring of 12 identical subunits as shown by the presence of a 12-fold rotational symmetry, following self-rotation and power spectrum analysis. The cavity accommodates neatly the X-ray crystal structure of the *N. gonorrhoeae* pilin subunit. The structure of the *N. meningitidis* PilQ was further resolved at 12 Ǻ [72]. 

More recently, the studies of Jain *et al.* have shown that secretin complexes contain previously unidentified large and flexible extra domains that probably stabilize or assemble Tfp. In this study, secretin complexes of *N. gonorrhoeae* show a double ring structure with a 14–15-fold symmetry in the central ring, and a 14-fold symmetry of the peripheral ring with seven spikes protruding [73]. In contrast, the spikes were absent and the peripheral ring was partly or completely lacking in *N. meningitidis* secretin complexes. When present, the ring has a 19-fold symmetry. Using NMR, Berry *et al.* derived the structures of the periplasmic domains from *N. meningitidis* PilQ secretin [74]. In addition, the structure of the entire PilQ dodecamer was also revealed by cryo-EM as a “cage-like” structure that enclosed a large cavity (~55 Å in internal diameter at its largest extent). The entire PilQ assembly that spans the periplasm has been reconstructed and NMR chemical shift mapping was used to generate a model for the PilP:PilQ interacting complex, adding further information to the three-dimensional reconstruction of the complex obtained previously at low resolution by TEM [75]. 

The structures of several other pilus-associated proteins have been resolved. These include solution structures of folded domains of the PilP lipoprotein [76], the high-resolution crystal structure of PilW, the partner lipoprotein of PilQ from *N. meningitidis* [77], as well as the three-dimensional EM structure of the integral PilG inner membrane protein multimer [78]. Using X-ray crystallography, Helaine and colleagues reported that PilX shows the α/β- roll fold shared by all pilins and that PilX protein co-localizes with Tfp [79]. The ultrastructure of the *N. gonorrhoeae* PilT, a biological motor required for the retraction of Tfp, was examined by freeze-etch EM, which revealed a 115 Ǻ outer diameter and 15–35 Ǻ inner diameter ring [80]. This study also showed that the ultrastructures of gonococcal PilT and a PilT from *Aquifex aeolicus* (a chemolithotrophic bacterium) are similar to type II and type IV secretion ATPases.

### 2.2. Opacity-Associated Proteins: Opa and Opc

The most abundant protein adhesins in the outer membrane (OM) are Opa and Opc. The colony opacity-associated (Opa) protein (an eight-stranded β-barrel structure with four surface-exposed loops, 27–31 kDa), previously known as PII or class 5 protein, is commonly expressed in both meningococci and gonococci. The name “opacity” was coined for colonies that showed an opaque appearance when viewed on a microscope with oblique sub-stage lighting [81]. A single meningococcal strain can harbour 3–4 *opa* genes (*opaA*, *opaB*, *opaD* and *opaJ*) [82,83], whilst up to 11 *opa* genes can be expressed in gonococci at separate loci throughout the genome [84]. The commensal strains *N. subflava*, *N. mucosa* and *N. sicca*, were shown to harbour one *opa* gene, whereas two *opa* genes were found in *N. flava* and in *N. lactamica* [85,86,87].

In early structural analyses using rules derived from porin crystal structure and the conservation of sequence homology within transmembrane β-strands, Malorny *et al.* generated a two-dimensional structural model of Neisserial Opa that presented four surface-exposed loops [88]. Circular dichroism has been used to determine the structure of refolded and purified opacity proteins OpaJ129 and OpaB128 derived from *N. meningitidis* strain H44/76, and this indicated a high content of β-sheet conformation, which is consistent with the previously proposed topology model [89]. Although the crystal structure of Opa remains unsolved, Opa is structurally similar to the Neisserial surface protein A (NspA), for which a crystal structure was reported in 2003 [90]. Little is known about the role of NspA as an adhesin. Expression of Opa protein exhibits significant phase and antigenic variation. Comparison of amino acid sequences of different Opa proteins show that Loops 2 and 3 are hyper-variable (containing HV1 and HV2 regions), Loop 1 (proximal to the *N*-terminus) is semi-variable and Loop 4 is constant. Antigenic variation is attributable to intra-or inter-genomic recombination [91]. Sequence diversity in the loop regions is responsible for conferring specificity for host cell receptors. Amongst gonococci, an estimated 77% of Opa diversity is due to recombination within the same isolate, 16% is due to imported genes from other isolates and only 7% is due to *de novo* mutation [92]. Phase variation of Opa expression is determined by the variable number of pentameric coding repeats (5'-CTCTT-3') at the 5' gene region encoding the leader peptide [93]. The different number of repeats can lead to frame-shifting by slipped-strand mis-pairing during DNA replication and leads to high frequency of phase variation (~1 × 10^−3^ per cell per generation) [94]. The rate of gonococcal Opa phenotype transition was estimated to be about 2 × 10^−3^ per colony forming unit per generation [95]. Phase variation of gonococcal Opa is also affected and regulated by promoter strength, and results in expression levels ranging from no Opa to multiple Opa [96]. A similar translational control mechanism has been reported for meningococcal Opa protein [85] and certain types of Opa protein are more predominant in invasive isolates due to their high virulence properties [97,98]. *N. lactamica* Opa proteins are more similar genetically to meningococcal Opa (70% of genetic relatedness). Expression of Opa proteins has been demonstrated in some commensal strains, including *N. lactamica*, *N. subflava* and *N. flavescens* and share a phylogenetical cluster association different from a pathogenic cluster [87].

By contrast, the Opc protein (27–31 kDa), formally known as 5C protein, is only expressed in *N. meningitidis* [99]. Opc protein is encoded by a single gene, *opcA*, whilst the *opcB* is a “pseudogene” found on a second locus in both meningococcal and gonococcal genomes [99]. In a study of *opcA* gene in commensal strains, only two out of 13 screened *N. polysaccharea* strains harboured the *opcA* gene, which shared 93% homology to gonococcal *opcA* gene [100]. However, significant difference was observed within the region encoding the most surface-exposed loops and there is no evidence of Opc protein expression by these commensal strains with *opcA* genes. Although amino acid sequence variation amongst different Opc proteins is limited, the levels of Opc protein expression are phase variable. This phase variation is due to the transcriptional regulation of a homopolymeric, variable-length and polycytidine (poly-C) tract, which is located at the promoter region of the *opc* gene [101]. The crystal structure of OpcA has been determined to 2.0 Ǻ resolution and shows that this adhesin adopts a 10-stranded β-barrel structure; protruding above the predicted membrane surface are extensive loop regions of dramatically different conformation with a high degree of flexibility [102,103,104].

### 2.3. Classical Monomeric Autotransporters: App and MspA/AusI

*Neisseria* can export monomeric autotransporter adhesins, App and MspA/AusI, through a type Va secretion system [105,106,107]. The IgA protease of *N. gonorrhoeae* was identified as the first example of a classical autotransporter [108]. The modular structure of autotransporter proteins contains three parts: (1) a *N-*terminal signal peptide; (2) a secreted passenger domain and (3) a *C*-terminal translocator domain. The *N-*terminal signal peptide can target the unfolded passenger domain, crossing the inner membrane to the periplasmic space via Sec machinery. Next, the *C-*terminal translocator domain folds into a β-barrel (also named as β-domain) followed by insertion into the OM, a process facilitated by an OM multi-protein machine called the Bam protein complex [109]. The integral translocator domain in the OM has a central hydrophilic channel, which can act as a pore and is essential for transportation of the passenger domain to the cell surface. During transportation across the OM, the passenger domain can undergo periplasmic and extra-cellular folding via interaction with other proteins, such as chaperones. On secretion, the passenger domain can finally exert its biological functions [109].

The highly conserved Neisserial App (Adhesion and penetration protein, 160 kDa, the product of the *app* gene) shares a high degree of homology to Hap (Haemophilus adhesion and penetration protein, the product of the *hap* gene) in *Haemophilus influenzae* [105]. The crystal structure of *Haemophilus* Hap has been solved [110] and it is defined as a prototype self-associating autotransporter (SAAT), which can mediate bacterial cell-cell adhesion and facilitate bacterial aggregation and biofilm formation. X-ray crystallography (2.2 Å resolution) determined the crystal structure of the *Haemophilus* Hap_S_ passenger domain, which harbours the SAAT domain. Structural analyses shows that Hap forms intercellular multimeric complexes that are required for bacterial cell-cell interaction and microcolony formation [110]. In the Neisserial App protein, a serine protease motif in the β-domain pilots the autoproteolytic activity and subsequent secretion of the passenger domain. Mutation of Ser^267^ has been shown to abolish the autocatalytic cleavage and therefore proves that the catalytic triad in the passenger domain—His^115^Asp^158^Ser^267^—which is also present in Hap protein of *H. influenzae* and in nine other autotransporter proteases, contributes to autoproteolysis [106]. All *Neisseria* species possess the *app* gene and the meningococcal App protein amino acid sequence shares ~95% and 73% identity with *N. gonorrhoeae* and *N. lactamica* App, respectively.

MspA (meningococcal serine protease A, 157 kDa) or AusI (autotransporter serine protease I) was designated due to its homology to App (33% identity), IgA1 protease (36% identity) and other autotransporters [107]. The *mspA* gene is not present in all meningococcal strains and its expression is also phase variable. Similar to App, MspA also has a catalytic triad (His^100^Asp^135^Ser^241^) in the predicted passenger domain [107]. To our knowledge, no structures have been solved by crystallography/NMR for MspA.

### 2.4. Trimeric Autotransporter Adhesins: NadA and NhhA

The trimeric autotransporter adhesin (TAA) family of secreted Gram-negative OM proteins are organised in obligate homotrimers. The structures of TAAs show a simple “head-stalk-anchor” organisation. Trimerization of the head (passenger domain) is required for stability and adhesive ability to host tissue [111,112], the stalk projects the head beyond the membrane and the β-barrel anchor (*C-*terminal translocator domain) is responsible for secreting both head and stalk components. In contrast to classical autotransporters, typical autocatalytic cleavage does not occur in TAAs, so the functional passenger domain remains covalently attached to the anchor after being secreted [113]. The prototypical members of this family are the YadA protein of enteropathogenic *Yersinia* species [114,115] and Hia (*Haemophilus influenzae* adhesin) and Hsf (*Haemophilus* surface fibril) adhesins of *H. influenzae* [116,117]. NadA and NhhA are two TAAs that have been characterised in *Neisseria*. 

NadA (Neisserial Adhesin A, 38 kDa) is a member of the Oca (Oligomeric coiled-coil adhesins) family, and the protein shares ~32–34% homology to the UspA2 (ubiquitous surface protein A2) of *Moraxella catarrhalis* and the *Yersinia* YadA protein [118]. NadA was predicted to have three main domains: (1) a COOH-terminal anchoring domain (β structure), which is also necessary for auto-translocation to the bacterial surface; (2) a probably coiled domain with a leucine zipper, which might mediate dimerization and oligomerization; (3) a NH(_2_)-terminal globular head domain [118] that is involved in interactions with human cells. In addition, the apical region of NadA is predicted from EM and structure analysis to form a compact and globular domain [119]. The *nadA* gene is present in ~50% of meningococcal strains, but absent in both *N. gonorrhoeae* and *N. lactamica*. Interestingly, in a study of 154 carriage isolates, only 5.1% of the strains harboured the *nadA* gene [120]. 

NhhA (*Neisseria* hia/hsf homologue, 57 kDa) was first identified because of its similarity (47%) to the adhesin AIDA-I of *Escherichia coli* [121] and was later defined as a multifunctional TAA with close homology to the Hia and Hsf adhesins of *H. influenzae* [121,122]. It was suggested to name it Meningococcal surface fibril (Msf), since it is more similar (~74%) to *Haemophilus* surface fibril (Hsf) [123]. Its particularly short *C-*terminal translocation domain (the last 72 residues), defined as a minimal translocation unit, is capable of trimerization to form a complete β-barrel. NhhA is expressed in *N. meningitidis* and *N. lactamica*, but not in *N. gonorrhoeae* [122]. 

### 2.5. Two-Partner Secretion (TPS) System: HrpA and HrpB

The two-partner secretion pathway (TPS), composed of a large secreted protein (TpsA, typically > 100 kDa) with biological function and a transporter protein (TpsB, ~60 kDa) forming a β-barrel pore in the OM, is widely used by Gram-negative bacteria for exporting large proteins. In contrast to autotransporters that are encoded by single genes, TpsA and TpsB are encoded by two separate genes and function as “partners” [124]. The synthesized TpsA is transported to the periplasmic space via Sec machinery. In the following stage, the *N-*terminal TPS targeting domain (250-amino acid residues long) in the TpsA molecule can direct the TpsB protein in the OM, in order to transport TpsA across the OM via a type Vb secretion system [125]. The structure of the TPS transporter is very different to the translocator domain of the autotransporter system [126]. The prototype of this family is the filamentous haemagglutinin (FHA) of *Bordetella pertussis* [126,127], whose crystal structure has been solved [128]. Due to homology with FHA, TpsA and TpsB homologues in meningococci are designated as haemagglutinin/haemolysin-related proteins HrpA and HrpB, respectively [129]. *hrpA* (TpsA) genes are present in surveyed 822 meningococcal carriage strains and their encoded proteins can be classified into two groups [129]. *N. lactamica* only harbours genes encoding group II TPS system [129,130]. In a later study of 88 meningococcal disease isolates, the previous group II TPS system was further classified into systems 2 and 3. System 1 was ubiquitous, whereas systems 2 and 3 were found to be related to hyperinvasive clonal complexes [131]. In comparison to *hrpA* (TpsA) gene, the *hrpB* (TpsB) gene is highly conserved and essential for functional secretion of TpsA [129]. Examination of the specificity of the two TpsB transporter systems, TpsB1 and TpsB2, shows that the TpsB2 system is capable of transporting all types of TpsA domains, whereas TpsB1 is more specific to transportation of its cognate TpsA domains [132]. *N. gonorrhoeae* does not harbour a *hrpA* gene, but instead has a disrupted TpsB opening reading frame and thus it lacks a functional copy of a TPS system [131].

### 2.6. Other Neisseria Adhesins

The T-cell stimulating protein A (TspA, 92 kDa) of *N. meningitidis* was initially identified as a T- and B-cell stimulating antigen [133] and later shown to be required for efficient adhesion [134]. TspA shares homology with the FimV protein of *Pseudomonas aeruginosa* and was therefore classified as belonging to the FimV family, members of which are also present in other bacteria such as *Legionella pneumophila* and *Vibrio cholerae* [135]. TspA was found in all the meningococcal isolates surveyed, with >85% identity. Similar TspA amino acid sequences were also identified in *N. polysaccharea*, but not in *N. lactamica* or *N. gonorrhoeae* [134]. 

Expression of fructose-1,6-bisphosphate aldolase (FBA, 38 kDa) is considered a requirement for efficient bacterial adhesion to host cells, although its role in this process in unknown. Moreover, FBA is present in both the cytoplasm and OM. The amino acid sequences of FBA are >99% identical between meningococcal isolates and also 70%, 67% and 65% identical to the class-IIB FBA from *Cupriavidus metallidurans*, *Xanthobacter falvus* and *Synechocystis* spp., respectively [136]. Glyceraldehyde 3-phosphate dehydrogenase (GapA-1, 37 kDa) is another enzyme with putative adhesin function, which is surface-located and highly conserved in meningococci (>97% identical) and also present in gonococci (99% identical to strain FA1090) and *N. lactamica* (90% identical to strain ST640) [137]. A putative ABC transporter component of a glutamate transporter operon, encoded by gene NMB1966 (29 kDa), has also been reported to mediate adhesion of meningococci to human bronchial epithelial cells [138].

The OmpA (outer membrane protein A)-like protein (23.4 kDa) of gonococci is homologous to OmpA of other Gram-negative species, including *E. coli*, *Salmonella*, *Yersinia* and *Pseudomonas* spp. (40–44% identity) [139]. The structure of *Neisseria* OmpA has not been elucidated. However, the *C-*terminal domain of the meningococcal RmpM (Class 4 protein, reduction-modifiable protein M) is homologous to the periplasmic, *C-*terminal domain of *E. coli* OmpA and shares ~35% amino acid sequence identity [140]. The crystal structure of this domain has been solved at 1.9 Ǻ and shows that the domain adopts a βαβαββ-fold. By analogy, structural information on both the transmembrane β-barrel and soluble periplasmic domain of OmpA from *E. coli* has been reported [141].

In a recent study, Hung *et al.* identified the meningococcal Adhesin Complex Protein (ACP, 13.3 kDa) as a new adhesin [142]. There are 31 different *acp* DNA alleles which encode 11 types of ACP in sequenced *Neisseria* isolates present in the original BIGS database of 205 genome-sequenced *Neisseriae* [143] and within a separate collection of 12 well-characterized meningococcal strains [142]. Meningococci express type I, II and III ACP with one or two amino acid differences (98–99% similarity). Commensal strains such as *N. lactamica*, *N. polysaccharea* and *N. sicca* also harbour the gene that encodes type I ACP. By contrast, gonococci carry the *acp* genes encoding two types of ACP, which show 94% identity to meningococcal ACP [142]. The structures of TspA, FBA, GapA-1, NMB1966 and ACP have not been elucidated.

### 2.7. Other Neisseria Surface Structures Influencing Adhesion

Several other surface structures, although not functioning directly as primary adhesins, can play an accessory role during bacterial interactions or they can modulate the adhesion process itself. These structures include the polysaccharide capsule, lipo-oligosaccharide (LOS) and major OM porin proteins. In addition, many other *Neisseria* ligands can interact with human cell components, but are not classified as adhesins. 

#### 2.7.1. Polysaccharide Capsule

The polysaccharide capsule is an important virulence factor for meningococci, enabling this pathogen to avoid complement-mediated killing and phagocytosis. In contrast, gonococci and the well-studied commensal strain, *N. lactamica* do not have capsules [144]. The highly hydrated capsule most likely ensures meningococcal survival in aerosol droplets (*i.e.*, avoiding desiccation), which is important for survival in fomites and for person-to-person transmission [145]. Many colonizing meningococci are non-capsulated [146], whereas those causing invasive disease are almost invariably capsulated. Additionally, the phase-variable on/off expression of capsule can influence the interactions between meningococci and host cells [147].

To evade host immune recognition, meningococcal capsules also exhibit characteristic antigenic variation and immune mimicry. According to serological reaction to capsules, meningococci are classified into 12 serogroups [148], amongst which serogroup A, B, C, Y and W cause the majority of invasive meningococcal disease. The genes encoding the biosynthesis of sialic acid (NANA, 5-*N*-acetyl-neuramic acid) for serogroups B, C, Y and W are similar. This similarity can facilitate horizontal gene exchange and result in capsule switching between these invasive serogroups [149]. However, sialic acids are present commonly on human cells and therefore meningococci can avoid recognition by immune molecular mimicry. The most studied example is the serogroup B meningococcal capsule, which contains a α(2-8)-linked polysialic acid that is structurally identical to glycosyl residues of human neural cell adhesion molecule (NCAM) [150,151]. 

Solution conformations of the group B polysaccharide have been analyzed by DQF-COSY and pure absorption 2D NOE NMR [152] and essentially complete ^1^H-NMR conformations for serogroup A, C, W and Y polysaccharides also have been determined [153]. High resolution-magic angle spinning (HRMAS) NMR spectroscopy has been used to determine the exact structures of serogroup A capsular polysaccharide expressed on meningococci [154]. Structure of the serogroup X polysaccharide has been described using a combination of ^13^C, ^1^H and ^31^P-NMR spectroscopy and total correlation spectroscopy (TOCSY) and ^1^H-^13^C heteronuclear single quantum coherence (HSQC) [155]. More recently, quantification of serogroup X capsular polysaccharide by proton qNMR has been reported [156]. 

#### 2.7.2. Lipo-Oligosaccharide (LOS)

LOS is composed of a lipid A, an inner and outer core oligosaccharide and is structurally distinct from lipopolysaccharide (LPS) of Gram-negative enteric bacilli due to the lack of a repeating polysaccharide O-side chain [157]. Both *N. meningitidis* and *N. gonorrhoeae* harbour the *lgt* genes encoding LOS with high genetic diversity, whereas the *lgt* gene is not carried by all the commensal strains [158,159]. LOS exhibits antigenic variation, which is mostly due to phase variation of the related gene expression [160]. Sialylation of LOS in serogroups B, C, W and Y enables meningococci to mimic host cell surfaces that also express sialic acid [161]; as a consequence, the organism becomes more resistant to antibody and complement-mediated killing and phagocytosis [162,163].

Structural characterisation of *Neisseria* LOS has a long history, but detailed descriptions are outside the remit of this current review. A variety of biochemical and biophysical techniques have been used for LOS structural determination: these include methylation analysis, specific degradations (e.g., mild acid hydrolysis) and high-pH anion-exchange chromatography of underivatised oligosaccharides with reverse-phase high performance liquid chromatography; pulsed amperometric detection (HPAE-PAD) of oligosaccharides (OS), fluorophore-assisted carbohydrate electrophoresis monosaccharide composition analysis; 1D 500-MHz ^1^H-NMR as well as nuclear Overhauser effect experiments; 2D NMR methods [double quantum filtered COSY (DQF-COSY), delayed COSY (D-COSY), homonuclear Hartmann-Hahn spectroscopy (HOHAHA) and pure-absorption 2D NOE NMR]; mass spectrometry techniques (positive ion fast atom bombardment; Fourier transform ion cyclotron resonance mass spectrometry (FTICR-MS); electrospray ionization, collision-induced dissociation, and multiple step) [164,165,166,167,168,169,170,171,172,173,174]. Structural information on LOS has been recorded for meningococci [168,172,174,175,176,177], gonococci [178,179] and several commensals (*N. canis*, *N. perflava*, *N. subflava*, *N. flava*, *N. cinerea*, *N. flavescens*, *N. caviae*, *N. sicca*) [180,181,182].

#### 2.7.3. Porin (Por)

Porins comprise up to 60% of the proteins present in the *Neisseria* OM. Most *Neisseria* spp*.* express only one Por, whereas meningococci have both PorA (~41–42 kDa) and PorB (~34 kDa), which allow selective passage of cation and anion across the cell membrane respectively [183]. Recently, PorB was also shown to mediate cation transport [184]. *N. gonorrhoeae porB* gene exists as two alleles that encode either PorB.1A or PorB.1B protein [185]. Gonococci also have a *porA* gene, but it is present as a pseudogene due to frame shift or promoter mutation [186]. The porin of *N. lactamica* and *N. polysaccharea* are identical and show similarity to meningococcal PorB and gonococcal PorB.1A and PorB.1B (~35–37 kDa) [187,188]. 

Porin structural analysis has mainly been directed towards PorA and PorB; early three-dimensional model structures were derived by comparison of PorA and PorB *Neisseria* sequences with known *E. coli* porin structures [187]. Despite the low sequence identity with *E. coli* porins, *Neisseria* spp. porins assemble into a model of the 16-strand β-barrel fold characteristic of porins. Meningococcal PorB forms stable trimers [189] and the X-ray crystal structure was determined to 2.3 Ǻ resolution, which identified three putative solute translocation pathways through the channel pore [190,191]. Recently, the gonococcal PorB.1A was analysed by X-ray crystallography in the presence of phosphate and ATP and a detailed structure solved at a resolution of 3.3 Å [192]. Surprisingly, no complete X-ray crystal structure has been provided for meningococcal PorA; only structural analyses on the binding complexes formed between different monoclonal antibodies and different peptide epitopes corresponding to porin subtype variants have been reported [193,194]. Even less is known of porin structure in other *Neisseria*: however, ribbon diagrams comparing the *N. lactamica* porin protein with related meningococcal PorB have been published, but structural examination showed that variation in *N. lactamica* porin was less than that observed in the meningococcal porin [188].

## 3. Mechanisms of *Neisseria* Adhesion with Host Cells

**Figure 1:**
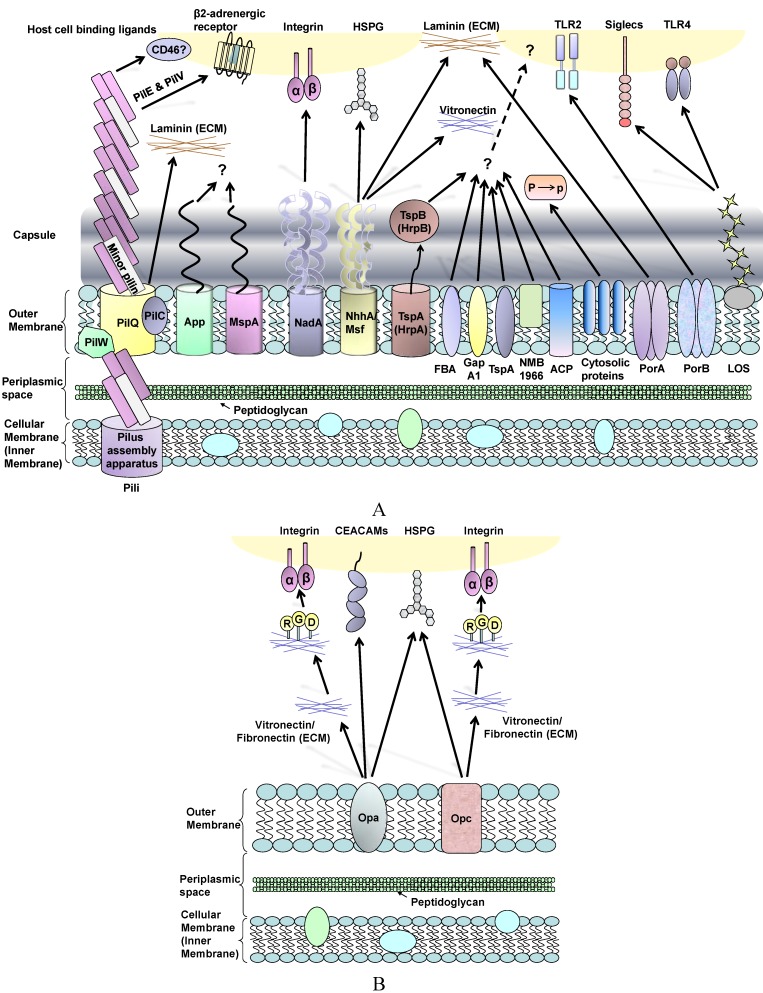
(**A**) Schematic review of *Neisseria meningitidis* surface molecules and their interactions with human host cell binding ligands. Many of these structures are absent in gonococci, whereas OmpA is specific to this organism (see text). Plasminogen-binding cytosolic proteins include enolase, DnaK and peroxiredoxin; P to p denotes conversion of plasminogen to plasmin. Far less is known about surface structures in commensal *Neisseria*; (**B**) Interactions between *Neisseria* Opa and Opc proteins and human host cells involves multiple binding ligands. Opa proteins are expressed by meningococci and gonococci, whereas Opc is only expressed by meningococci. Opa and Opc-mediated binding events are more efficient in the absence of capsule.

The pathogenic *Neisseriae* share similar surface structures that are involved in adhesion, but they also display subtle and disparate mechanisms for interacting with the host. Here, we review the adhesion process based on the different histology of host cells and sites of infection, and highlight the bacterial ligand-host cell receptor interaction mechanisms that may dictate tissue tropisms (Figure 1).

### 3.1. Neisseria meningitidis Adherence to Nasopharyngeal Epithelial Cells

The natural habitat of the meningococcus is the surface of the human nasopharyngeal mucous membrane and transmission is mainly through close contact. Increased bacterial cell surface and surface hydrophobicity have been suggested to correlate with increased levels of association with airway epithelial cells [195]. Once meningococci make contact with the human nasopharyngeal epithelium, there are two major steps involved in bacterial adhesion: “initial adhesion” [55] followed by “intimate adhesion” [196].

Pili are the most important adhesin during the “initial” binding of capsulated (and indeed non-capsulated) meningococci to epithelial cells [197]. After initial attachment, pili retract to bring meningococci closer to the host cells and subsequently the bacteria lose piliation [198]. The pilus tip-located protein PilC has been postulated to play an essential role in the adhesion process [199]. PilT protein is essential for the ATP-dependent pilus retraction machinery, which powers bacterial twitching motility [38,200,201]. PilT also mediates loss of piliation and progression from a localised to a diffuse pattern of adherence to epithelial cells [202]. This diffuse pattern is visible as bacteria spreading onto the apical surface of the cells and forming a monolayer, single-cell in depth. Moreover, PilT-driven pilus retraction requires down-regulation of PilC1 [64]. The nature of the target receptor for meningococcal/gonococcal pilus remains elusive. It has been suggested that pili facilitate adhesion by binding to a membrane-located human complement regulatory protein, CD46 (Membrane Cofactor Protein) [203]. Johansson *et al.* also demonstrated that transgenic mice expressing human CD46 are susceptible to meningococcal infection [204]. However, contradictory studies showed no correlation between the levels of CD46 isoform expression and pilus-mediated adherence of gonococci [205] and that gonococcal pili adherence to urogenital epithelial cells could occur via a CD46-independent process [206].

Other adhesins such as App [106], NadA [207], NhhA [122], HrpA-HrpB [129], FBA [136], GapA-1 [137], TspA [134] and ACP [142] have all been shown to mediate adhesion of capsulated bacteria. The involvement of these surface structures has been inferred from infection studies with deletion mutants and the use of purified ligands in cell binding assays. These adhesins are considered to be involved in the initial adhesion process. Regarding known host receptors, NadA binds human β1 integrins [208] and NhhA binds laminin and heparin sulphate [122], whereas host cell receptors recognising the other adhesins have not been identified.

A role for plasminogen binding to the bacterial surface could be a contributory mechanism that enables meningococcal interactions with the nasopharyngeal mucosal epithelium. The mucous barrier contains extracellular matrix (ECM) components such as glycoproteins and proteoglycans, which are also found within submucosal tissue and as components of the basal laminae of epithelial and endothelial cell barriers. Meningococcal surface-associated components have been reported to bind plasminogen, which is then converted to enzymatically active plasmin. Significantly, this binding/activation event is not inhibited by the presence of capsule [209]. The meningococcal components identified were enolase (a 46 kDa glycolytic enzyme), DnaK (a 67 kDa heat shock protein) and peroxiredoxin (a 24 kDa protein that contains domains found in reducing enzymes and electron transporters) [209]. Plasminogen conversion to plasmin on the meningococcal surface leads to plasmin-activated collagenase activity. It is possible that this activity enables the bacterium to degrade ECM components within the mucosa, thereby allowing pathogen access to epithelial cells. In addition, meningococcal survival is favoured by the proteolysis of complement and secretory IgA antibodies that are found on mucosal surfaces. Thus, plasminogen binding contributes to meningococcal adherence and survival during colonisation of the host.

Following initial adherence, the presence of capsule can sterically hinder the contribution of surface-expressed, OM-located adhesins that are required for “intimate” adhesion. In order to enable intimate contact, activation of the CrgA regulatory protein (that represses capsule synthesis and export) down-regulates capsule expression [196]. However, the role of CrgA in down-regulation of capsule and pilus is controversial, since other studies of the CrgA regulatory protein [210,211] could not reproduce the findings of Deghmane *et al.* [196]. Without the hindrance of capsule and following pilus retraction, “intimate” adhesion involves a different repertoire of adhesins, of which Opa and Opc proteins are the most dominant [212]. PilT also appears to be essential for inducing intimate attachment of meningococci, as demonstrated by infection studies *in vitro* with T84 intestinal epithelial cells [202].

Opa protein shows a tropism for human receptors, which fall into two major categories. Most Opa proteins bind to members of the human carcinoembryonic antigen (CEACAM) receptor family, whereas a smaller number bind to heparan sulphate proteoglycan (HSPG) [213,214], integrins, ECM proteins such as vitronectin and fibronectin [215,216] and saccharides [217]. The biology of the CEACAM receptor family is outside the scope of this review, but the interested reader is directed towards the review of Gray-Owen and Blumberg [218]. Within the CEACAM family, there is one secreted and 11 cell surface glycoproteins and of these, CEACAM 1, CEACAM3, CEACAM5 (CEA, the first member of the family [219]) and CEACAM6 bind to *Neisseria* Opa proteins [220,221,222,223,224,225,226,227,228]. CEACAM proteins are expressed on apical surfaces of polarized cells, whereas HSPGs are not. CEACAM1 is expressed by epithelial and endothelial cells, neutrophils and lymphocytes; CEACAM3 is exclusively expressed by neutrophils; CEACAM5 is found in epithelial cells of the gastrointestinal and urogenital tracts and CEACAM6 is expressed in different organ epithelial cells and by neutrophils. Most of the studies of Opa-CEACAM interactions have been done *in vitro* using cell lines transfected to express human CEACAM proteins. Although Opa interactions are most efficient in the absence of capsule, Opa-mediated interactions of capsulated bacteria have been reported [229], but only to human cells expressing a high density of CEACAM1 [222]. Thus, it is possible that capsulated meningococci can penetrate epithelial cell barriers without a need to down-regulate capsule expression.

The inflammatory *milieu* and viral infection can also influence Opa-mediated interactions of meningococci with epithelial cells [229,230]. Meningococcal adherence is increased when cells are exposed to IFNγ (but not TNF-α or IL-1β), and this is due to Opa expression acting in tandem with IFNγ to drive *de novo* synthesis of CEACAM1 on the surface of epithelial cells. It has also been suggested that innate immune responses are down-regulated by Opa-CEACAM interactions. In a study with primary pulmonary epithelial cells, meningococcal Opa-CEACAM1 interactions lead to reduced Toll Like Receptor (TLR)2-initiated, NF-κB-dependent inflammatory responses. The inhibitory mechanism involves tyrosine phosphorylation of the ITIM (immunoreceptor tyrosine-based inhibitory motif) of CEACAM1 and by recruitment of the phosphatase SHP-1, which negatively regulates TLR2-dependent activation of the phosphatidylinositol 3-OH kinase-Akt kinase pathway [231]. 

Meningococci and gonococci that express Opa appear to bind to the *N-*domain region of CEACAM proteins, within the 1–108 amino acid region [221,225,227]. Opa recognition is a function of the non-glycosylated CEACAM backbone [222,232], and the binding surface is composed of the β-strands C’’, C’, C, F and G and appears to require Tyr^34^ and Ile^91^ exposed residues [233]. Variation of amino acid residues 27–29 found in different CEACAMs directs differential binding of Opa to CEACAM1, 5 or 6 [227]. Specific conformational interactions between the hypervariable (HV1, HV2) regions within Opa loops appear to be necessary for binding to CEACAM [234] and despite high levels of sequence variation in Opa HV regions, the binding sites for CEACAM do show significant conservation. In studies with *N. meningitidis* strain H44/76, which expresses OpaA and OpaJ binding to CEACAM1 and OpaB and OpaD binding to CEACAM1 and CEACAM5, respectively, a comparison of the binding of hybrid variants of Opa HV1 and HV2 demonstrated the presence of a conserved binding motif in the HV2 region of all four Opa proteins [235]. This motif consists of Gly^172^, Ile^174^ and Gln^176^.

Opc acts independently in adhesion and invasion of meningococci into epithelial and endothelial cells [236] and the host cell receptors that recognise Opc include HSPG [237], integrin and ECM proteins [238,239]. Opc is an important adhesin that binds to the cytoskeletal protein α-actinin of epithelial and endothelial cells after bacterial invasion [240]. Furthermore, a low expression level of Opc can allow more Opa-dependent invasion of primary nasopharyngeal cells [241]. However, it remains unclear how Opa and Opc proteins interact with each other during adhesion, or indeed invasion. 

Little information is known regarding the role of other adhesins for intimate contact. Capecchi *et al.* demonstrated that a non-capsulated MC58 NadA deletion mutant (∆NadA) had a reduced ability to invade into Chang epithelial cells compared to wild-type MC58 [207], but there were no differences in terms of total association. GapA-1 has been shown to play a role in the association of non-capsulated MC58 to Hep-2 cells [137]. Without the presence of the capsule, pili, Opa and Opc, an MC58 ACP deletion mutant (∆ACP) showed reduced association with Chang epithelial cells compared to its parent strain [142]. Whether these adhesins work in concert or independently remains to be confirmed. 

Following initial attachment, successful colonisation of epithelial cells by meningococci and adaptation to the nasopharyngeal niche requires bacterial aggregation for micro-colony formation and resistance to biophysical clearance mechanisms. Initially, PilX enables bacterial aggregation before Tfp-mediated adherence to the nasopharyngeal epithelium [68]. Tfp also form bacterial aggregates associated with host cells, and the Tfp-mediated adhesion process triggers cortical plaque formation within epithelial cells. The components of the cortical plaque include CD44v3 (a HSPG), EGFR (epidermal growth factor receptor, a receptor tyrosine kinase), CD44/ICAM-1 (adhesion molecules) and f-actin and ezrin (a component that links the membrane components to the actin cytoskeleton) [242]. In response to cross-talk with *Neisseriae*, the clustering of membrane proteins that correlate with cytoskeletal rearrangements has been demonstrated on host cells. This process might enable more avid binding of bacterial ligands with host cell receptors and result in greater resistance to detachment by shear stress [242]. Tfp-mediated adhesion can also contribute to a phase-variable post-translational modification, in which addition of phosphoglycerol to pili allows detachment of bacteria from the bacterial aggregates and dissemination to other sites [243]. In addition, gonococcal Tfp and PorB.1B have been reported to perturb the trafficking of lysosome-associated membrane protein (Lamp)1 in A431 cells (derived from an epithelioid carcinoma) or T84 colorectal epithelial cells by triggering separate and distinct Ca^2+^-dependent exocytotic processes [244,245]. This results in bringing Lamp1 to the cell membrane, where it becomes accessible to cleavage by IgA protease.

Nasopharyngeal carriage of meningococci that is established after successful colonisation, can last, *in extremis*, up to 10 months [246], suggesting the possibility of biofilm existence as a mechanism to explain persistence. A biofilm is defined as an “intimately associated bacterial community included within an exopolymeric matrix” composed of lipid, polysaccharides, proteins, membrane vesicles, cell debris and extracellular DNA, with the material predominantly produced by the organism [247]. The biofilm forming process involves bacterial adherence, aggregation and microcolony formation [247,248] and this microbial community can resist mechanical forces and protect bacteria from host immunity. However, there is limited clinical evidence of meningococcal biofilm formation [249]. In the laboratory, biofilm formation on an abiotic polystyrene surface was a feature of ~30% of meningococcal carriage isolates, compared to ~13% of disease-causing isolates [250]. Capsulated isolates show a reduced biofilm-forming capability on abiotic surfaces [251] and pili are not required for biofilm formation. However, the importance of pili-associated proteins in biofilm formation is supported by evidence that a *pilQ* mutant formed biofilms that were thinner than those formed by wild-type bacteria [250]. PilX has been shown also to support biofilm formation indirectly [251]. Both capsulated and non-capsulated meningococci formed biofilms on monolayers of human bronchial epithelial cells [252]: thus, the limitation of capsulated meningococcal biofilm formation on abiotic surfaces suggests the importance of host cell factors in this process and/or a general phenomenon dependent on hydrophobic interactions with cell surfaces [253]. Additionally, surface-located HrpA plays a role in meningococcal biofilm formation on human bronchial epithelial cells [254]. Although incompletely understood, meningococcal biofilm formation depends on the functional properties of extracellular DNA, which serves to support initial attachment to the substratum and also to mechanically stabilize the growing biofilm structure [253]. The DNA for initial attachment is released during normal growth by the actions of membrane-bound enzymes such as the lytic transglycosylase A/B (MltA/B) and *N-*acetylmuramyl-l-alanine amidase (AmpD), which are involved in normal physiological cell wall recycling [255]. However, the roles of other adhesins in meningococcal biofilm formation have not been investigated.

### 3.2. Neisseria Gonorrhoeae Adhesion to the Urogenital Tract

#### 3.2.1. Gonococcal Infection of the Human Lower Reproductive Tract

In contrast to meningococci, gonococci do not express capsule or Opc protein. The major adhesins of gonococci include pili, LOS, Opa and porin. The interactions of gonococci with host epithelial cells depend on cell histology, site of infection and gender. For example, Opa^+^ and Opa^−^ strains are distributed in different areas of the urogenital tracts. Clinically, Opa^+^ strains are related to more asymptomatic but invasive disease, whereas Opa^−^ strains cause more symptomatic but non-invasive disease [256].

Gonococci primarily infect and colonise the exposed mucosal epithelium of the human reproductive tract and subtly different mechanisms of bacterial interaction have been observed between sexes. Infection of the male urethra is believed to occur in a two-step process that involves the interactions of gonococcal surface ligands with specific host cell receptors. Adherence to the urethral mucosal epithelium is mediated initially by the binding of pilus to the I-domain region of α_1_β_1_ or α_2_β_1_ integrins [256]. Next, the gonococcus-bearing integrin forms a transient interaction with the asialoglycoprotein receptor (ASPG-R), which leads to a tight interaction between the gonococcal and urethral cell plasmalemma membranes. In addition, ASPG-R also recognises and binds the terminal galactose of gonococcal LOS [256,257]. The evidence for this mechanism comes from infection studies using primary male urethral epithelial cells, whole urethra tissue and from examination of clinical samples of urethral exudates. 

Surprisingly, the mechanism of gonococcal transmission from partner-to-partner has not been studied in great detail. However, piliated gonococci do show higher levels of adherence than non-piliated bacteria to human sperm [258] and a binding interaction between LOS and ASGP-R on the surface of sperm cells has been reported [259]. Gonococcal adherence has no deleterious effects on sperm motility or viability [260]. How gonococci detach from sperm and/or seminal fluid proteins during ejaculation and attach to partner urogenital or ano-rectal epithelial tissue is not known. Moreover, how gonococci establish an association with the mucosal epithelium in the healthy female genital tract during transmission is still unclear, particularly the mechanisms by which the organism successfully competes with an established microflora and bypasses the environmental and innate immune barriers. 

In the female urogenital tract, gonococcal first-contact is with mucosal epithelial cells of the cervix and an elegant model describing the process has been described recently by Edwards and Butler [261], using information from studies of gonococcal-positive clinical biopsies and bacterial infection of human primary cervical epithelial cells (abbr. pex) *in vitro*. The infection process is clearly influenced by the menstrual cycle and involves the alternative complement pathway. Pex secrete all of the proteins that constitute the pathway and complement protein C3, in particular, is critical for the gonococcal infection process. During the luteal phase and menses, C3 is released into the environment and is converted to C3b during gonococcal infection. C3b binds to the gonococcal surface and is cleaved to form inactivated iC3b. Clinically isolated gonococci contain iC3b on their surface [262], which binds naturally to the I-domain of CR3. The human cervix expresses high levels of CR3 (the α_m_β_2_ integrin also known as CD11b/CD18) [263] and hence gonococci have been observed to co-localize with CR3 *in vivo* [263]. A glycan moiety on pilus also allows pilus binding to the CR3 I-domain and draws the gonococcus to the epithelial cell surface [264,265]. In addition, a tight association between gonococcus and receptor is effected through the interactions of iC3b and porin (PorB.1A or PorB.1B), which also bind to the CR3 I-domain [266]. CR3 engagement also results in the release of gonococcal components such as phospholipase D, which is suggested to aid colonization [261]. Furthermore, factor H has been reported to facilitate gonococcal adherence to eukaryotic cells by forming a “bridge” with CR3 [267]. 

ASPG-R receptors are present on mucosal surfaces of the female urogenital tract, but do not appear to mediate LOS-dependent gonococcal adherence to pex. In addition, Opa proteins do not mediate gonococcal association with primary epithelial cells [264,268,269]. Nevertheless, data from *in vitro* studies do suggest that LOS and Opa may be involved in adherence of gonococci with immortalised and/or transfected cell lines. Mutation of LOS encoded by *lgtF* was shown to reduce gonococcal interactions with ME-180 cervical carcinoma cells *in vitro*, suggesting a possible role of particular LOS types for association [270]. Gonococci expressing OpaA have been shown to associate tightly with HeLa cells [271], possibly through HSPG interactions [272]. Opa interactions with HSPG require vitronectin and fibronectin ECM components, which function as a “molecular bridge” to mediate adherence with an integrin co-receptor (α_v_β_1_, α_v_β_3_, α_v_β_5)_ [215,216,273]. The Opa-CEACAM interaction(s) does not require ECM component bridges and is a direct protein-protein binding event [222,233], although it is still unclear whether CEACAM-gonococcus interactions occur during cervical infection *in vivo* [261].

The ability of gonococci to cause asymptomatic infection raises the speculation that gonococci can persist in the female reproductive tract in biofilms. Gonococci can form biofilms in continuous-flow chambers *in vitro*, both on glass surfaces and on cultured human primary urogenital tract epithelial and cervical epithelial cells [274]. In the study of Greiner *et al.* the entire cervical epithelial cell culture was covered by a gonococcal biofilm, without obvious damage to the cells, even after eight days. Moreover, *in vivo*, there are reports of gonococcal biofilm formation on cervical cell biopsy specimens obtained from patients with gonococal infection and of bacterial presence on intrauterine devices [275,276]. OM blebbing appears to be essential for matrix formation and for stabilising the gonococcal biofilm structure [276]. However, little is known regarding the roles of surface adhesins/other molecules in biofilm formation and a detailed review of their composition and the metabolic phenotype of gonococcal biofilms is available elsewhere [277]. Within a biofilm, the interactions of gonococci with host cells can range from weak, leading to bacterial detachment, to persistent, which is a consequence of extracellular polymer binding. Subsequent to gonococcal adhesion, pathogen-induced activation of signalling pathways and membrane/cytoskeletal rearrangements occur in the host cells, leading to invasion. The mechanisms involved in gonococcal invasion of cervical epithelial cells are outside the scope of this review, but outlined in the comprehensive review of Edwards and Butler [261].

#### 3.2.2. Ascending Gonococcal Infection

Gonococcal colonization of the cervical mucosal epithelium provides a platform for the organism to colonize exposed mucosal surfaces in the upper reproductive tract. The exact mechanisms used by gonococci to disseminate from the lower reproductive tract to the upper reproductive tract are not known. Fluid movement of planktonic bacteria is likely to be involved, possibly after detachment from motile ascending sperm and/or release from an established biofilm in the cervix. Ascending infection of gonococci into the body of the uterus can cause endometritis, which is possibly the transition zone between uncomplicated cervicitis and complicated PID. Gonococci have been shown to adhere to and invade transformed human endometrial adenocarcinoma cells (HEC-1B cells) [278] and primary endometrial cells [279] *in vitro*. Interestingly, vaginal *Lactobacillus* spp. can inhibit gonococcal interactions with endometrial cells [280] and *Lactobacillus jensenii* releases a surface protein, as yet uncharacterised, which is considered to mediate this process [281]. Gonococcal pili and Opa co-operate during adherence to and invasion of HEC-1B cells [282]. This co-operative interaction was reported to co-localise with ASPG-R and CEACAM receptors on epithelial cells of the endometrium [283], suggesting that expression of these host cell receptors is possibly important for ascending infection. In addition, OmpA protein has been shown to mediate gonococcal adhesion to human cervical carcinoma and endometrial epithelial cells *in vitro* [139].

Gonococcal infection can ascend further to involve the fallopian tubes (FT) and cause salpingitis and other complications of PID [284]. Gonococcal adherence to and invasion into FT epithelial cells were demonstrated using a FT explant organ culture model [285,286]. Specific attachment of gonococci to non-ciliated FT epithelial cells is probably mediated by pili and Opa [287]. Piliation has been reported to enhance gonococcal adherence to FT explant epithelium within 3 h of infection *in vitro*, but thereafter does not offer gonococci any advantage in colonizing the epithelial cell surface [288]. However, pilus does appear to inhibit several key elements of the initial inflammatory response, thus facilitating sustained infection of the FT [288]. Different Opa-expressing variants of *N. gonorrhoeae* show differences in attachment to FT mucosa and damage to mucosal cells [289]. Gonococcal LOS and Opa were shown to interact with TREM-2 (triggering receptor expressed on myeloid cells-2) on genitourinary and FT epithelial cells [290]. Binding of gonococcal Opa to human CEA on transgenic mouse urogenital cells has been shown to trigger expression of the transforming growth factor receptor CD105, which is a member of the transforming growth factor-β1 receptor (TGFβ1R) family and affects integrin expression. The resulting activation of integrin expression could enhance cell adhesion and suppress epithelial cell exfoliation, which is used by the host as a mechanism to protect the mucosa [291]. In addition, gonococci adherent to FT epithelial cells *in vitro* have evolved a mechanism to protect host cells from undergoing TNF-α-mediated apoptosis (which is responsible for FT mucosal epithelium damage) and this modulation of the host innate response probably contributes to establishment of infection [292].

Expression of PorB.1A has been observed to correlate with severe DGI [185,293,294]. PorB.1A, but not PorB.1B, mediates a phosphate-sensitive invasion mechanism [266] and a specific binding to human heat shock glycoprotein (Gp96) and the scavenger receptor (SREC) on epithelial cells during gonococcal invasion has been demonstrated [295]. 

### 3.3. Adherence of Neisseria spp. to Endothelial Cells

Survival of *N. meningitidis* in the blood is determined by virulence factor expression and host innate and adaptive immune mechanisms. Even if meningococci successfully enter the blood via micro-vessels or capillaries, they can be killed by host effector immune cells responding to the induction of a transient bacteraemia. However, if meningococci survive in the blood as a result of ineffective innate and adaptive immune responses they can multiply rapidly, disseminate to multiple organs and cause overwhelming septicaemia and/or meningitis. 

**Figure 2:**
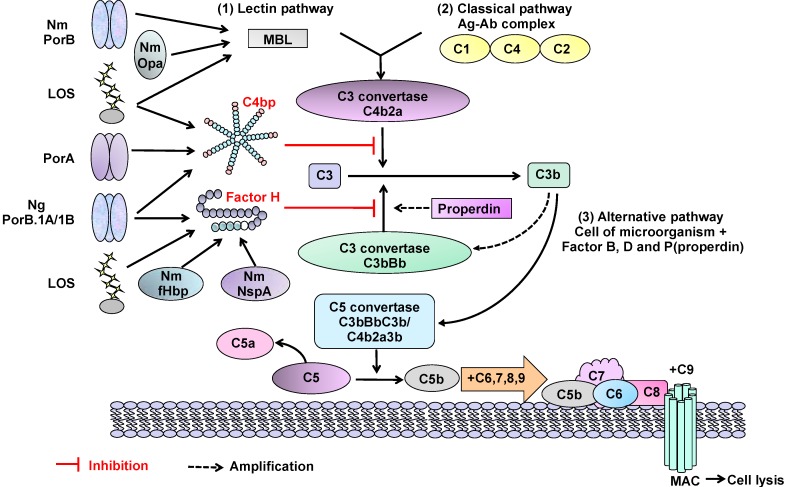
Interaction of Neisserial surface molecules with the complement system. The interactions of *Neisseria* molecules with components of the human complement system can have inhibitory or stimulatory effects. (i) Lectin pathway activation: binding of meningococcal (Nm) Opa and PorB proteins by MBL (mannose-binding lectin) is a possible mechanism that accelerates complement activation and increases bacterial killing. The core structure of LOS can also bind MBL, but binding is reduced following LOS sialylation; (ii) Classical pathway down-regulation: Nm PorA, gonococcal (Ng) PorB.1A and PorB.1B and LOS can also bind C4bp (complement regulatory protein C4b-binding protein), which is the main inhibitor of the classical pathway; (iii) Alternative pathway down-regulation: Nm fHbp and NspA, LOS and Ng PorB.1A and PorB.1B bind to complement-inhibitory regulator serum factor H and down-regulate the alternative pathway. Gonococcal strains expressing PorB.1A bind factor H (and/or C4bp) avidly, whereas strains expressing PorB.1B bind factor H weakly. However, sialylation of LOS increases binding of PorB.1B to factor H. The binding of some strains expressing PorB.1B to C4bp can occur regardless of LOS sialylation.

Prevention of phagocytosis and binding of complement and antibody is crucial for meningococcal survival in the blood. Capsule and LOS are the most essential virulence factors that enable survival [296] and both are also associated with resistance to complement-mediated killing [297,298]. Shedding of excessive OM forming “blebs” [299] also allows meningococci to divert antibodies and complement away from the bacterial surface. LOS can cause coagulopathy, endothelial disruption and circulatory collapse [300,301]. To evade complement-mediated killing, meningococci can interact with host complement regulatory proteins to down-regulate the complement cascade (Figure 2). For example, PorA protein has been shown to bind C4bp (complement regulatory protein C4b-binding protein), the main inhibitor of the classical pathway [302]. Moreover, meningococcal factor H binding protein (fHbp) and NspA bind to factor H on human complement and down-regulate the alternative pathway [303,304]. Gonococci can also resist serum complement-mediated bactericidal activity and this mechanism is dependent on the binding of human C4bp and factor H by porins and sialylated LOS. Within gonococcal PorB.1A, surface-exposed Loop 1 is necessary for binding to C4bp, whereas Loops 5 and 7 of PorB.1B are believed to form a negatively charged C4bp-binding domain. The binding site of PorB.1A for factor H has been identified on Loop 5 [305,306,307]. Binding of activated vitronectin by meningococcal NhhA/Msf has been reported to down-regulate the terminal phase of the complement pathway [123] and NhhA has also been shown to prevent phagocytosis and complement-mediated killing [308].

Conversely, the binding of several *Neisseria* components can play a role in controlling bacterial growth in the blood. Binding of meningococcal Opa and PorB proteins by the the serum collectin mannose-binding lectin (MBL) is a possible mechanism that accelerates complement activation and increases bacterial killing [309]. The core structure of gonococcal LOS can also bind MBL, but binding is reduced following LOS sialylation [310] (Figure 2).

Rapid multiplication of meningococci in the blood promotes vascular colonisation, *i.e.*, an adherence of bacteria to luminal endothelial cells. This involves several steps: (1) initial adhesion; (2) proliferation, aggregation and formation of micro-colonies; and (3) dissemination. Pili, Opa and Opc remain the most important adhesins for pathogenic *Neisseria* to interact with endothelial cells. Recently, an *in vivo* model of human dermal microvessels introduced into SCID/Beige mice by xenografting of human skin has been used to examine meningococcal interactions with vessel endothelium *in situ* [311]. Type IV pili were the primary mediators of association with dermal microvessels and association was accompanied by a potent inflammatory response, the recruitment of inflammatory cells, and local vascular damage resulting in *purpura*. Importantly, this animal model replicates clinical presentation in human infection. Moreover, meningococci lacking pili were non-adhesive and non-inflammatory.

Opc is the most effective protein in increasing meningococcal interaction with human umbilical vein endothelial cells (HUVECs) [212], whereas, in contrast, an OpaB-expressing strain showed lower association with HUVECs compared to Chang epithelial cells [212]. Binding of meningococcal Opc to HUVECs is mediated through a tri-molecular complex that includes serum vitronectin and fibronectin connected to integrins in the presence of the amino acid sequence arginine-glycine-aspartic acid (RGD) “bridge” [238]. Similar findings using human brain microvascular endothelial cells (HBMEC) have also been reported [239]. The integrin receptors for vitronectin and fibronectin are α_v_β_3_ and α_5_β_1_, respectively. Sulphated tyrosines of activated vitronectin have been identified as the targets of Opc binding for adherence to and subsequent invasion of HBMEC [312]. CEACAM1 is also found on endothelial cells and therefore a likely target for Opa-binding: indeed, as a consequence of meningococcal (and indeed gonococcal) stimulation of TNFα during intravascular dissemination, CEACAM1 expression is found in increased levels on endothelial cells [228,313]. As expected, binding of Opa-expressing *Neisseriae* to endothelial surfaces is dramatically increased.

Other adhesins are involved in endothelial cell interactions, although the recognition receptors are not known. FBA and GapA-1 mediate association of meningococci to HBMEC in a capsule-independent manner [137]. Hung *et al.* also demonstrated that meningococcal ACP mediates adhesion to HUVECs, independent of capsulation [142]. MspA can mediate adhesion to human bronchial epithelial cells and HBMEC, but this study was done using *E. coli* expressing MspA rather than with Msp-expressing meningococci [107]. Comparative studies of MC58ΔMspA and wild-type bacterial interactions would confirm if MspA is important for meningococcal adhesion to human cells. 

The ability to resist shear stress in the blood flow is a requirement for meningococcal adherence to endothelial cells. Soyer and Dumenil have defined shear stress as the “tangential force exerted per unit area by a fluid moving near a stationary wall” [314]. PilV was shown to trigger plasma membrane reorganisation and to recruit cholesterol to form filopodia in endothelial cells, a mechanism that leads to enhanced micro-colony cohesion. These lipid rafts enable meningococci to form membrane protrusions on the endothelial surface and to secure the micro-colony from shear stress [315]. Recently, meningococcal PilE and PilV have been reported to bind the β2-adrenoceptor/β-arrestin pathway in order to enable meningococci to cross the brain microvasculature endothelium [316]. However, meningococcal adherence to epithelial cells does not involve the PilE/PilV: β2-adrenoceptor/β-arrestin binding events [317]. PilX plays a role in conformational changes of meningococcal Tfp during signalling to endothelial cells [318].

As one would expect from the absence of significant case reports of DGI, little is known regarding the interactions of gonococci with vascular endothelial cells. Virji *et al.* demonstrated that gonococcal pili are important for adhering to HUVECs and that pili variants showed differences in the extent of adhesion [319]. Both purified gonococcal and meningococcal PilC proteins were shown to bind HUVECs [320]. In addition, different gonococcal Opa proteins display different affinities in binding to CD66 (CEACAM) receptors, which could contribute to conferring cell tropism [226,321]. 

### 3.4. Interactions of Neisseriae with Immune Effector Cells

Once in the blood, meningococci (and gonococci during disseminated infection) interact with immune effector cells, including neutrophils, monocytes, macrophages, dendritic cells and B and T cells. There is a significant amount of knowledge regarding *Neisseria* interactions with neutrophils, which are facilitated by the down-regulation of sialic acid on LOS and capsule [322]. Originally, Opa proteins were called “leucocyte association proteins” [323] and hence the majority of Opa-expressing *Neisseria* interact with neutrophils. As with non-myeloid cells, Opa mediates “intimate” interactions with neutrophils, and some protein variants are involved in adherence, whereas others promote phagocytosis. Recently, the Tfp of pathogenic *Neisseria* have been shown to mediate adhesion to the uropod of polarized neutrophils. This binding was PilC1/PilC2-dependent for meningococci, but not for gonococci [324]. Gonococci within gonorrhoeal exudates are observed attached and within neutrophils and bacterial attachment is effected by the co-operative interactions of Opa, pili, porin and LOS [325]. LOS modifications that are known to influence *Neisseria* interactions with host cells, including neutrophils, are phosphoethanolamine (PEA) substitution on lipid A or oligosaccharide, and sialylation of the oligosaccharide terminal Galb1-4GlcNac epitopes. For further details regarding the interactions between pathogenic *Neisseria* and neutrophils, including Opa-CEACAM1, 3 and 6 interactions, phagocytic uptake of both meningococci and gonococci and stimulation of bactericidal oxidative burst response, the reader is referred to the excellent reviews from Sadarangani and colleagues [326], Johnson and Criss [325] and Criss and Seifert [327]. 

Studies with purified, but non-native conformational PorA, PorB, Opa and Opc from meningococci show that Opa induces the strongest T-lymphocyte proliferative responses *in vitro* [328]. However, conflicting data have been obtained using Opa in its native conformation, as presented in OMV and on whole bacteria. For example, the activation and proliferation of CD4+ T-lymphocytes to various stimuli *in vitro* is abrogated by treatment with Opa-containing OMV from meningococci and the effect is due to Opa binding to CEACAM1 [329]. Furthermore, Opa present on whole gonococci has been reported to suppress CD4+ T-lymphocyte activation and proliferation through the same CEACAM1 binding event [330]. A possible mechanism to explain Opa-mediated inhibition of CD4+ T-lymphocytes has been described by Sadarangani *et al* [326]: normally, T-cell activation occurs when antigen presented with MHC Class II by antigen-presenting cells, is bound by the T-cell receptor, leading to Src kinase stimulation, which phosphorylates cytoplasmic tyrosine residues of CEACAM1 and activates downstream signalling pathways. However, binding of Opa to CEACAM1 recruits the tyrosine phosphatases SHP-1 and SHP-2, which dephosphorylate CEACAM1, the T-cell receptor and intracellular signalling pathway proteins, leading to inhibition of T-cell activation. It is not clear whether Opa-induced activation or inhibition of T-cell responses occurs *in vivo*; despite the fact that antibodies to Opa are observed after natural meningococcal infection or vaccination (albeit with wide variation in antibody levels) [331], the potential ability to down-regulate immune responses is not an attractive quality for a vaccine antigen. It has also been suggested that gonococcal Opa-CEACAM1 interactions inhibit B lymphocyte antibody production by inducing cell death [332], although (Opa-dependent) gonococcal association *per se* could be a significant inducer of cell death, rather than a consequence of the specific ligand-host cell receptor interaction.

Although not important as adhesion events that enable bacterial colonization, activation of immune effector cells has been reported for other *Neisseria* ligands: purified recombinant PorA protein can activate monocyte-derived dendritic cells and direct T-cell differentiation towards a Th2 type response [333], NadA can stimulate inflammatory cytokine production by human monocyte-macrophages [334] and NhhA can induce apoptosis of macrophages [335]. Recently, whole meningococci have been reported to bind to galectin-3 and increase interaction with phagocytic cells [336]. LOS sialylation can lead to increased susceptibility to host clearance mechanisms: in this context, the interaction between LOS and sialic acid-binding immunoglobulin-like lectins (siglecs), which are expressed predominantly in the cells of the haemopoietic and immune systems, are important. Sialoadhesin (Sn, siglec-1) and siglec-5 bind to terminal NeuAcα2,3Gal on meningococcal LOS and have been shown to contribute to increased macrophagocytosis of bacteria [337]. In addition, the class A scavenger receptor on macrophages can bind to meningococcal proteins NMB1220, NMB0278, and NMB0667 [338] and enhance bacterial uptake. Serum C-reactive protein can also bind to phosphorylcholine on meningococcal pili and increase opsonophagocytic uptake by macrophages and neutrophils [339].

However, there is a significant amount of knowledge regarding immune effector cell activation by *Neisseria* occurring through the repertoire of known host cell TLRs. TLR recognition of *Neisseria-*associated molecular patterns (NAMPs) plays a critical role in host innate and inflammatory defence against infections caused by these organisms. Several NAMP-TLR interactions have been reported and most studies have focused on LOS/LPS and porin B interactions with both myeloid and non-myeloid cells. In general, cell activation in response to Gram-negative LPS depends on sequential LPS interactions with LPS-binding protein (LBP), CD14, MD-2 and TLR4. Endotoxin lipid A interacts directly with MD-2 to activate TLR4 and there is a correlation between variations in the structure of the lipid A, which can influence the binding affinity of meningococcal LOS for human recombinant MD-2 and the subsequent activation of TLR4 that leads to cytokine production by human macrophages [340,341]. Recently, three-dimensional molecular simulations have been used to identify and characterize the interactions between TLR4-MD-2 with meningococcal and *E. coli* LPS molecules, which control TLR4 dimerization. Significantly, differences in the surface structure of the MD-2-LPS complex correlate with the ability of variably acylated LPS molecules to activate the innate immune system via TLR4 [342]. Conversely, inhibition of inflammation can be observed following a LPS-TLR4 interaction, but this is dependent on negative regulation by CEACAM1. Treatment of neutrophils with LPS has been reported to trigger the formation of a TLR4-pSyk-CEACAM1 complex, and subsequent recruitment of SHP-1 to the CEACAM1 ITIMs inhibits the production of IL-1β by the inflammasome [343]. This mechanism bears an interesting similarity with Opa-CEACAM down-regulation of other innate immune responses.

Activation by non-LPS NAMPs has also been reported to occur through TLR4 (Table 2), although this interpretation must be tempered by consideration of the presence of contaminating LPS in any of the preparations. Generally, bacterial proteins/lipoproteins are believed to interact with TLR2, or TLRs other than TLR4. The meningococcal porin B protein has been extensively studied and binds directly to a TLR2-TLR1 heterodimer [344,345], which mediates cell activation and protects cells from apoptosis. PorB can act through TLR2 as a B-cell mitogen [346] and porin-induced dendritic cell activation is MyD88 and TLR2 dependent [347].

**Table 2:** Role of TLRs in the recognition of *Neisseria* molecules.

*Neisseria* ligand	TLR, cell expression and host response	Reference
*Neisseria* LPS	TLR4	See text for details
*Neisseria* PorB	TLR2	See text for details
Meningococcal NhhA	TLR4 activation in macrophages	[348]
Product of meningococcal NMB1468 (Ag-473)	TLR4 activation in bone marrow-derived dendritic cells	[349]
Meningococcal penicillin-binding proteins	TLR4 activation of dendritic cells	[350]
Meningococcal recombinant NadA(Δ351-405)	Binds to monocyte HSP90 and forms a transducing complex of HSP90/HSP70/TLR4	[351]
Meningococcal CPS	Macrophage recognition via TLR2- and TLR4-MD-2 pathways.	[352]
Meningococcal membrane-associated proteins (LPS^−^ background)	Activate TLR2-CD14 in monocytes/macrophages	[353,354]
Gonococcal Lip (H.8) protein	Stimulates human endocervical epithelial cells in TLR2-dependent manner to secrete cytokines	[355]

The *N. lactamica* PorB is also a TLR2 ligand, but its binding specificity for TLR2 is different from that of meningococcal PorB, and it induces lower inflammatory responses [356]. Recently, the resolved crystal structure of PorB (2.3 Å resolution) suggests that TLR2-mediated recognition may be initiated by a non-specific electrostatic attraction [191]. Toussi and colleagues identified amino acid residues present in Loops 5 and 7 of meningococcal PorB that influenced TLR2 interactions, but conceded that the surface-exposed loops were not uniquely responsible for PorB-TLR2 interactions, and proposed the presence of a hypothetical “TLR2-binding signature” within the loops [357].

### 3.5. Interaction of Neisseria with the Meninges

The exact mechanism by which *Neisseria* spp. enter the CSF-filled subarachnoid space (SAS) compartment to induce meningitis is not known. The blood-brain barrier (BBB) in this context is less relevant than the blood-CSF barrier (BCSFB), as bacteria do not seem to invade the brain parenchyma. One route of entry for bacteria into the CSF could be through the choroid plexus, but although meningococci adhere to the endothelium of choroid plexus capillaries [358], it is unclear how bacteria would penetrate the choroid plexus epithelium. Meningococci were not detected inside or between choroidal epithelial cells [358], suggesting that bacteria are unable to penetrate the tight junctions of the choroidal epithelium. A recent study of intranasally infected mice, has suggested that meningococci can pass directly from the nasopharynx to meninges through the olfactory nerve system in the absence of bacteraemia [359]. However, bacteraemia is normally a prerequisite for meningococcal invasion into the CSF and it is more likely that thin-walled veins in the SAS are the primary routes of entry for bacteria into the SAS [360]. Furthermore, it is probable that bacteria enter the CSF and SAS through the same pathways that polymorphonuclear leucocytes take from the blood to the CSF. Meningococcal pili have been shown to maintain adherence to cerebral endothelial cells under high flow conditions *in vitro* [361] and PilQ and PorA proteins have been identified as ligands for the 37/67-kDa laminin receptor on the surface of rodent and human BMEC [362]. Subsequently, internalised meningococci might reach the meninges via transcytosis or disruption of intercellular junctions [51]. 

Although the molecular basis of the interactions that occur between *Neisseria* spp. and human epithelial, endothelial and immune effector cells have been studied in detail, little is known on the events that occur within the SAS after bacterial entry. The interactions of *Neisseria* spp. with cells of the leptomeninges (*i.e.*, the arachnoid mater, pia mater and trabeculae that traverse the SAS) play a fundamental role in meningitis. Animal models have been described for studying the pathological consequences that occur following the interactions between bacteria and the meninges, but there are significant anatomical differences between the human leptomeninges and the meninges in experimental animals [360]. Arachnoid trabeculae are absent in the mouse leptomeninx, the SAS is restricted in rodents and *zonula adhaerens* are present between rat arachnoid cells, whereas desmosomal junctions are found in humans. Moreover, the culture of primary human leptomeningeal cells as a surrogate *in vitro* model is unreliable [363]. Thus, information on the nature of the *Neisseria* ligands mediating binding to the leptomeninges has been collected from *in vitro* studies using frozen human brain tissue samples and cultured primary meningioma cells.

Meningioma cells are derived from benign tumours (meningiomas) of the human leptomeninges and they share the same morphology, cytological structure and expression of cell markers (desmosomal desmoplakin, cytokeratin, vimentin and epithelial membrane antigen) as normal leptomeningeal cells [363,364,365]. The capsulated and piliated phenotype of *N. meningitidis*, which is normally isolated from the CSF [299], shows a specific predilection for binding to the leptomeninges and meningeal blood vessels but not to the cerebral cortex [366]. Meningococci adhere similarly to meningioma cells in culture [366]. Notably, *N. gonorrhoeae* and the commensal *N. lactamica* also adhere to the leptomeninges and meningioma cells [367], which is unsurprising, since leptomeningitis caused by these organisms has been reported to occur as a rare event during disseminated infection [368,369]. 

The major surface ligand that mediates adherence of meningococci to the leptomeninges and meningioma cells is the pilus [366]. Although the levels of adherence are similar for bacteria expressing either Class I or Class II pili, variation in Class I pilin influences the ability of piliated meningococci to interact with leptomeningeal cells, which has also been observed with epithelial and endothelial cells [370,371]. Meningococci expressing Pil_Ib_^+^ show significantly reduced association to both fresh human brain sections and meningioma cells, compared to Pil_Ia_^+^ expressing bacteria. How pilin variation influences the adherence of meningococci to leptomeningeal cells is unclear, but variable expression of the PilC1 protein is not a factor and neither the presence nor the absence of capsule interferes with Pil_1a_-mediated adherence [366]. It is possible that post-translational modifications of pilin molecules [372,373], or the ability to form bundled pili [374], are important. Expression of Opa protein does not influence the ability of capsulated meningococci expressing high affinity Pil_1a_ to adhere, but Opa protein expression does increase the adherence of meningococci that express the low adhesive pilin Pil_1b_ [366]. In contrast, Opc protein does not play a role in meningococcal adhesion to meningioma cells. The role of other meningococcal adhesins for interacting with meningeal cells is not clear. In one study, decreased association of a TspA mutant of MC58 with meningioma cells has been reported, but the data were not shown [134]. More recently, ACP protein has been shown to mediate adherence of capsulated meningococci to meningioma cells *in vitro* [142]. In addition, during meningococcal meningitis, innate recognition of both LPS and non-LPS modulins is dependent on the expression of non-TLR pattern recognition receptors on cells of the meninges [375]. 

## 4. Conclusions

Bacterial adhesion to eukaryotic cells is a critical step in colonization, whether we consider the natural commensal flora or the introduction of competing species with pathogenic potential. A great wealth of knowledge is now available on the biology of *Neisseria* adhesins and the target human cell receptors as well as the molecular bases of their interactions. Despite a vast literature, our understanding of *Neisseria* adhesion to human cells and the host response to bacterial contact is still incomplete. In this review we have focused specifically on the structure and biology of *Neisseria* adhesins and the nature of the *Neisseria* ligand-host cell receptor interactions, but we have not discussed in detail the changes that occur to both the bacterium and the host cell as a consequence of adhesion. In-depth analyses of these changes are outside the scope of this review, but initial contact is known to stimulate global gene transcription for both *Neisseria* and host cells [376,377,378,379,380]. Membrane dynamics are altered with significant remodelling of the bacterial OM and host cell plasmalemma and the recruitment of host cell proteins. Contact also stimulates signalling pathways (signalosomes) in both the bacterium and host cell. Although these mechanisms remain incompletely understood, several excellent reviews that consider modulation of *Neisseria* and host cell biology after adhesion are recommended reading [51,163,381,382,383,384,385,386].

Why do *Neisseria* spp. have so many adhesins? Redundancy of adhesins may be a well-designed strategy for *Neisseria* to evade host immunity during either colonization or invasive disease. Generating the repertoire of known adhesins has also encouraged studies of their vaccine potential, although the (hyper-)variability and phase variation of many of these adhesins, e.g., pili and Opa proteins, likely precludes their use. However, meningococcal ligands that bind human cells but show greater sequence conservation, e.g., NadA and fHbp, are already included in the first generation of potentially “universal” vaccines, *i.e.*, Bexsero^®^ [387] and rLP2086 [388]. Other adhesins are also likely to be promising vaccine candidates that merit further study, in particular for the development of second-generation “universal” meningococcal vaccines and for re-energizing research into gonococcal vaccines, which has been intractable for decades. Understanding the biology of *Neisseria* adhesins could also lead to the development of anti-adhesin strategies, based on interrupting bacterial ligand-receptor interactions, but such strategies are still in their infancy. 

A perusal of Table 1 highlights our lack of knowledge on the interactions of commensal *Neisseria* organisms; in particular, how important are these organisms in niche occupation within the microbiomes of the respiratory (and possibly urogenital) tracts? Are these microbiomes stable or dynamic (due to continual horizontal intra- and inter-species gene transfer) and do commensal species use the same adhesion mechanisms, e.g., the detection of Opa-CEACAM binding in *N. subflava* (strain C450) [87]; or the expression of Tfp and other adhesins? Indeed, comparative genomics of commensal *Neisseria* spp. has revealed that these organisms share a large repertoire of virulence-associated alleles with meningococci and gonococci and also with other bacterial genera [130,389]. Moreover, amongst the commensal organisms, the largest set of virulence-associated alleles is found in *N. lactamica.* Thus, the genetic propensity for causing disease is available to commensals and diseases are occasionally reported (Table 1). Although commensal *Neisseria* contain genes encoding virulence factors, they probably do not normally express the profile of virulence-associated proteins required for infection. If commensal organisms and obligate pathogens share similar adhesins, would vaccines containing adhesin proteins influence the stability of the Neisserial commensal flora? Does this even matter? Indeed, how do adhesion mechanisms used by obligate pathogens allow these organisms to successfully compete with the commensal flora? Conversely, can commensal organisms be used to out-compete pathogens, as recently reported in studies of *N. lactamica* acquisition interfering with subsequent colonization by meningococci [390]?

**Figure 3:**
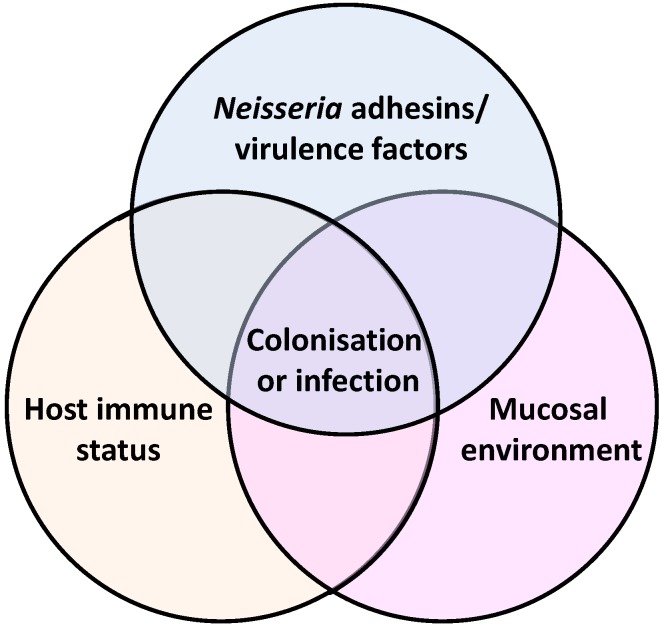
The interplay between *Neisseria* adhesins/virulence factors, host immune status and mucosal environment determine host colonisation or infection.

In conclusion, the evolution of specific *Neisseria* adhesins that enable primary colonization and subsequent maintenance of a commensal state or transient carriage is in many respects driven by a compliant host (Figure 3). The biological relevance of this compliance reflects homeostasis between the bacterial flora and host immune selection pressures. Nevertheless, what is certain is that adhesins expressed by the pathogenic *Neisseria* provide these organisms with the potential for host-to-host transmission, which is essential for survival of the species. Defining the roles played by *Neisseria* adhesins in pathogenesis present new opportunities for developing strategies to combat *Neisseria* infections.
